# *Culicoides* species community composition and infection status with parasites in an urban environment of east central Texas, USA

**DOI:** 10.1186/s13071-018-3283-9

**Published:** 2019-01-16

**Authors:** Estelle Martin, Elaine Chu, Phillip Shults, Andrew Golnar, Dustin A. Swanson, Jamie Benn, Dongmin Kim, Peter Schneider, Samantha Pena, Cassie Culver, Matthew C. I. Medeiros, Sarah A. Hamer, Gabriel L. Hamer

**Affiliations:** 1 Department of Entomology, Texas A&M University, College Station, Texas USA; 2 USDA-ARS Arthropod-Borne Animal Disease Research Unit, 1515 College Avenue, Manhattan, KS 66502 USA; 3 Department of Veterinary Pathobiology, Texas A&M University, College Station, Texas USA; 4 College of Veterinary Medicine and Biomedical Sciences, Texas A&M University, College Station, Texas USA; 5 Pacific Biosciences Research Center, University of Hawai‘i at Mānoa, Honolulu, Hawai‘i USA; 6 Department of Veterinary Integrative Biosciences, Texas A&M University, College Station, Texas USA

**Keywords:** Biting midges, Avian hosts, Onchocercidae, Haemosporida, Vector-parasite association, Integrative taxonomy

## Abstract

**Background:**

Despite their importance as vectors of zoonotic parasites that can impact human and animal health, *Culicoides* species distribution across different habitat types is largely unknown. Here we document the community composition of *Culicoides* found in an urban environment including developed and natural sites in east central Texas, a region of high vector diversity due to subtropical climates, and report their infection status with haemoparasites.

**Results:**

A total of 251 individual *Culicoides* were collected from May to June 2016 representing ten *Culicoides* species, dominated by *C. neopulicaris* followed by *C. crepuscularis*. We deposited 63 sequences to GenBank among which 25 were the first deposition representative for six *Culicoides* species: *C. arboricola* (*n* = 1); *C. nanus* (*n* = 4); *C. debilipalpis* (*n* = 2); *C. haematopotus* (*n* = 14); *C. edeni* (*n* = 3); and *C. hinmani* (*n* = 1). We also record for the first time the presence of *C. edeni* in Texas, a species previously known to occur in the Bahamas, Florida and South Carolina. The urban environments with natural area (sites 2 and 4) had higher species richness than sites more densely populated or in a parking lot (sites 1 and 3) although a rarefaction analysis suggested at least two of these sites were not sampled sufficiently to characterize species richness. We detected a single *C. crepuscularis* positive for Onchocercidae gen. sp. DNA and another individual of the same species positive for *Haemoproteus sacharovi* DNA, yielding a 2.08% prevalence (*n* = 251) for both parasites in this species.

**Conclusions:**

We extend the knowledge of the *Culicoides* spp. community in an urban environment of Texas, USA, and contribute to novel sequence data for these species. Additionally, the presence of parasite DNA (Onchocercidae gen. sp. and *H. sacharovi*) from *C. crepuscularis* suggests the potential for this species to be a vector of these parasites.

**Electronic supplementary material:**

The online version of this article (10.1186/s13071-018-3283-9) contains supplementary material, which is available to authorized users.

## Background

The family Ceratopogonidae (Diptera) includes the genus *Culicoides*, commonly known as biting midges or “no-see-ums”. *Culicoides* spp. are hematophagous pests and vectors of viruses, protozoans and filarial worms [1–3]. A major focus of research on *Culicoides* spp. is related to their roles as vectors of epizootic hemorrhagic disease virus (EHDV), bluetongue disease virus (BTV) and African horse sickness virus (AHSV) [3]. Beside their impact on livestock, *Culicoides* also transmit parasites to wildlife such as the avian trypanosome *Trypanosoma bennetti* [4] and *Haemoproteus* parasites [5, 6]; however, distributional patterns and infection prevalences of these parasites remain largely unknown.

One neglected area in the study of *Culicoides* spp. is their ecology in urban environments, where there is an interface with human populations. Prior studies have focused on specific species, such as pestiferous salt-marsh species [7] or species from specialized habitats such as zoos [8], and the vector-host-pathogen interactions in urban environments, could be of potential interest highlighting their potential as vectors of pathogens. Several species of *Culicoides* are known vectors of Haemosporida [9, 10], parasitic protozoans of amphibians, reptiles, birds and mammals [10]. These parasites can cause acute epizootic outbreaks that have severely altered avian communities by affecting long-term demographic processes such as reproductive rate and survivorship [10–12]. *Culicoides* are also involved in the transmission of filarial nematodes in the family Onchocercidae with infection report in the California quail, the American crow and the great-tailed grackle [13–15].

Recently, a high prevalence of haematozoan parasites was documented in a population of great-tailed grackles (*Quiscalus mexicanus*) in College Station, Texas, including *Heamoproteus*, *Plasmodium*, trypanosomes and filarial worms (Golnar et al., unpublished data). Previous work suggests that mosquitoes are not the vectors for these parasites and instead *Culicoides* are the more likely vectors [16]. Here, we document the *Culicoides* spp. inhabiting the urban environment of College Station, Texas, USA, and screen individuals for blood-borne parasites known to occur in local birds. The characterization of *Culicoides* species in this area where a high burden of avian parasites circulates provides an ideal opportunity to evaluate their potential role as vectors of haematozoan parasites.

## Methods

### *Culicoides* collection and identification

**Fig. 1:**
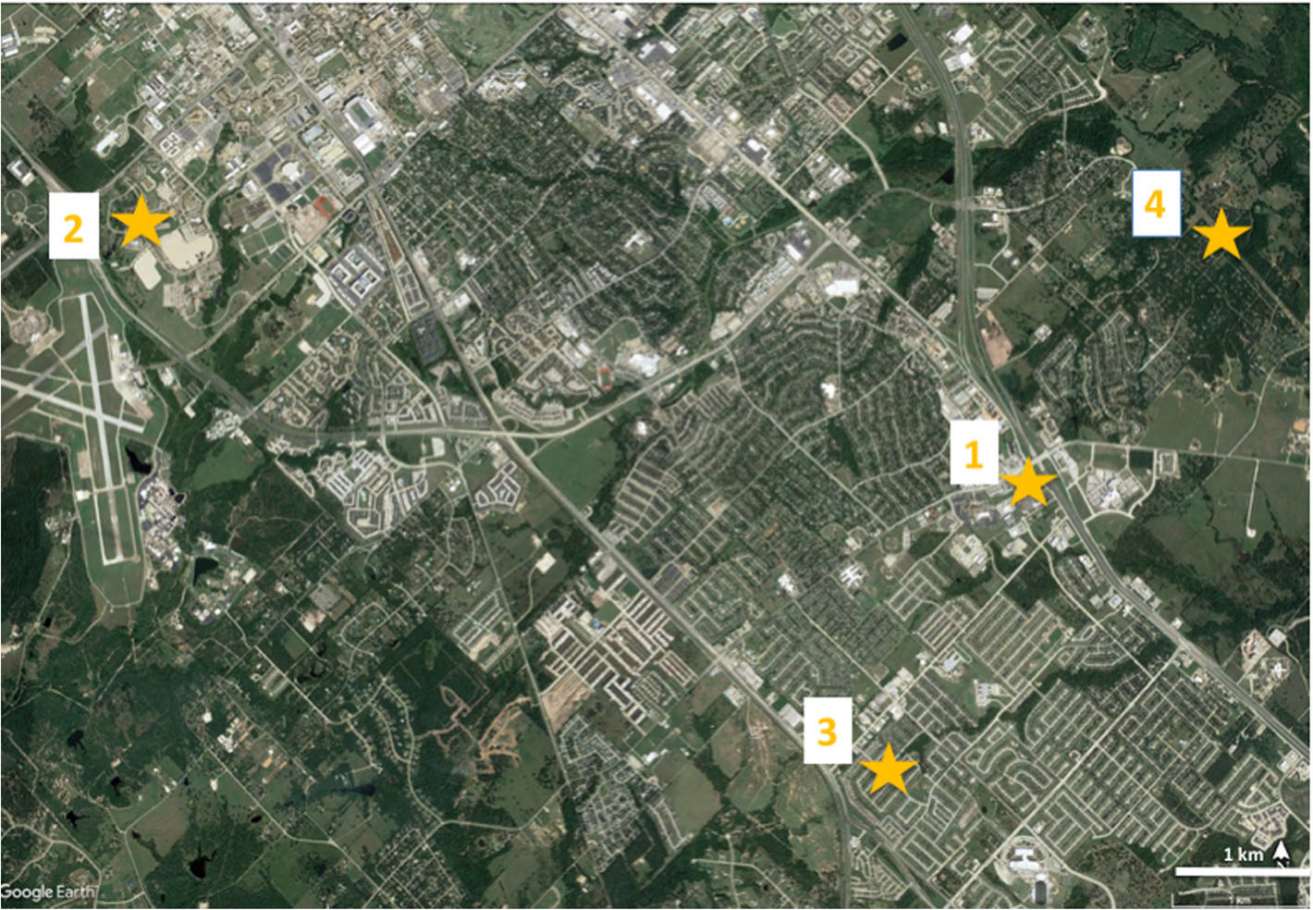
Trapping sites across College Station, Texas, USA. Blue boxes and site numbers are locations where traps were set

We collected *Culicoides* from College Station, Texas (30°37'40.7"N, 96°20'3.8"W) during the months of May and June, 2016. Centers for Disease Control and Prevention miniature light traps (CDC-LT, BioQuip model 2836BQ, with a 6 volt battery, USA) baited with 1.5 kg of dry ice were run from approximately 18:00 h to 8:00 h. Traps were placed in 4 localities: a grocery store parking lot (site 1), research park natural area (site 2), and two private homes in residential neighborhoods (site 3 and site 4) (Fig. 1). While site 1 and 3 are located in urban developed areas, sites 2 and 4 are located in urban natural environments with more vegetation in the surrounding landscape. In site 4, *Culicoides* traps were set in three locations, including one location next to a chicken coop. After collection, trap contents were chilled on ice for transport to the laboratory where specimens were identified to the species level based on wing pattern [17, 18] and stored at -20 °C until further processing.

We used an integrative taxonomic approach to identify *Culicoides*. Thirteen specimens were mounted on permanent slides to observe anatomic characteristics useful for species identification and were in parallel processed by a non-destructive DNA extraction method modified from the Gentra Puregene Kit (#D-5500A) (Gentra Systems, Inc., Minneapolis, USA). Whole specimens were added to individual Eppendorf tubes containing 100 μl of Cell Lysis Solution and 1 μl of Proteinase K and incubated overnight at 55 °C. Specimens were not crushed to preserve morphological features. The specimens were chilled at 0 °C for 20–30 min, after which 35 μl of 8.0 M ammonium acetate were added to each tube. The samples spun at 2200× *rpm* for 7 min in an Eppendorf 5424 Centrifuge. The supernatant was pipetted out of the tube and used to complete the DNA extraction protocol. Approximately 100 μl of 95% ethanol were added to the tube containing the exoskeleton to prevent further breakdown. The exoskeletons were separated into body regions (head, thorax, abdomen and wings) and transferred to 15.0% acetic acid for 10 min, 2-propanol for 10 min, and then 100.0% clove oil. Each body region of a single specimen was slide mounted in Canada balsam under its own coverslip. Slides were kept at 40 °C for 8–10 h and air dried for 72 h [19]*.* Slides were deposited in the Texas A&M University Entomology Museum under accession number 735. This method allowed for identification of specimens both molecularly and morphologically. Sequences of the cytochrome *c* subunit 1 (*cox*1) gene were obtained using the protocol described below.

All the remaining specimens (*n* = 238) were homogenized in a Mini-Beadbeater-96 (Bio Spec Products Inc., Bartlesville, OK, USA) with three 4 mm PYREX Solid Beads (Sigma-Aldrich, Inc., St. Louis, MO, USA) in 200 μl of Hank’s Buffer Salt Solution (ThermoFisher Scientific, Waltham, MA). DNA was extracted from 100 μl of the homogenized material (MagMAX CORE Nucleic Acid Purification Kit from Applied Biosystems, Foster City, CA). A PCR assay targeting a 710 bp region of the *cox*1 gene of invertebrates [20] was used for molecular identification of *Culicoides* species (Additional file 1: Table S1). The PCR consisted of 1.5 μl of genomic DNA, 0.5 μM of each primer, 12.5 μl of 1× Premix from the Epicentre Failsafe PCR purification kit (Epicentre Biotechnologies, Madison, WI, USA), and 1 unit of enzyme mix (total volume 25 μl). The thermal cycling profile consisted of denaturation at 95 °C for 3 min, followed by 35 cycles of 94 °C for 1 min, 45 °C for 1.5 min, 72 °C for 2 min, and a final extension step at 72 °C for 5 min. Amplicons were visualized on a 2% agarose gel. PCR products were purified using ExoSAP-IT PCR Product Cleanup (Affymetrix, Santa Clara, CA, USA) and bi-directional Sanger sequencing was performed (Eton Biosciences, San Diego, CA, USA). Forward and reverse sequence chromatographs were assessed for quality and sequences were aligned with sequences of *Culicoides* downloaded from NCBI’s GenBank database and analyzed using Geneious version 9.1.8 [21] using the Clustal-W method. Results from J-model test [22] indicated the best-fit selection model to run our analysis was GTR+G+I and therefore, maximum likelihood tree was constructed using Randomized Axelerated Maximum Likelihood (RAxML) with 1000 bootstrap replications. A phylogenetic tree was finalized using FigTree version 1.4.3 using a sequence of the *cox*1 gene of *Atrichopogon levis* (GenBank: KT092130.1) as the outgroup. In addition, fifteen previously published sequences of the *cox*1 gene of *Culicoides* species previously collected in Texas were included in this analysis (GenBank: KT794137.1; KT794138.1; KT794141.1-KT794144.1, KT794154.1; KT794155.1; KT794159.1; KT794161.1; KT794162.1; KT794164.1; KT794165.1; KT794167.1; KT794171.1) as well as 17 previously unpublished sequences (GenBank: MH751220, MH751222, MH751223, MH751224, MH751226, MH751227, MH751228, MH751235, MH751243, MH751244, MH751246, MH751248, MH751252, MH751258, MH751267, MH751273, MH751280) from specimens collected in Wisconsin, Wyoming and South Carolina and identified to species at the USDA-ARS Arthropod-Borne Animal Disease Research Unit in Manhattan, Kansas.

### Parasite testing

#### Filarial nematodes PCR assays

DNA extracted from each specimen was screened for the presence of filarial nematodes using a PCR assay amplifying a 580 bp region of the filarial nematode *18S* ribosomal gene (*18S* rRNA) ([23]; Additional file 1: Table S1). PCR cycling was performed at 94 °C for 2 min, 39 cycles of 94°C for 30 s, 57 °C for 30 s, 72 °C for 2 min, and a final extension step at 72 °C for 7 min. Samples that resulted in the successful amplification of the target pathogen were confirmed using a nested PCR targeting a 340 bp region of the *cox*1 gene ([16, 24, 25]; Additional file 1: Table S1). A touchdown cycling protocol was used and consisted of denaturation at 94 °C for 2 min; 8 cycles of 94 °C for 45 s, 51 °C for 45 s (reduced by 0.5 °C each cycle), and 72 °C for 1.5 min; followed by 25 cycles of 94 °C for 45 s, 45 °C for 45 s, and 72 °C for 1.5 min; and a final extension step at 72°C for 7 min. Amplicons were visualized and submitted for Sanger sequencing following the same protocol as above. Only those samples that resulted in amplification with the two PCR assays were considered positive in the determination of infection prevalence. When samples that screened positive using the *18S* rRNA gene PCR assay could not be confirmed using the nested *cox*1 gene PCR, samples where re-extracted from the original homogenate and subjected a second time to the *18S* rRNA gene PCR for confirmation.

#### Haemosporida PCR assays

Samples were screened for the presence of Haemosporida using a PCR assay targeting a 154 bp region of the *16S* ribosomal gene (*16S* rRNA) ([26, 27]; Additional file 1: Table S1). Reactions contained 1.0 μl of genomic DNA,0.4 μM of each primer, 1× Premix from the Epicentre Failsafe PCR purification kit, and 1 unit of enzyme mix (10 μl total volume). Cycling conditions included 2 min at 94 °C; 35 cycles of 94 °C for 50 s, 55 °C for 50 s, and 72 °C for 25 s; and a final extension step at 72 °C for 2 min. Samples resulting in amplification were confirmed by a nested PCR assay targeting a 552 bp fragment of the cytochrome *b* gene [26, 28, 29, 38] (Additional file 1: Table S1). In this nested PCR assay, 1 μl of genomic DNA, 0.2 μM of each primer, 0.1 μg/μl BSA, 1 × Premix from the Epicentre Failsafe PCR purification kit, and 1 unit of enzyme mix was used. Cycling conditions were 4 min at 94 °C; 35 cycles of 94 °C for 20 s, 49 °C for 10 s, and 72 °C for 45 s; and a final extension step at 72 °C for 3 min for the outer reaction and 94 °C for 1 min; 28 cycles of 94 °C for 20 s, 52 °C for 10 s, and 68 °C for 50 s; and a final extension step at 72 °C for 7 min for the inner reaction. Amplicons were visualized and submitted for Sanger sequencing following the same protocol as above. Only those samples with sequences from both the screening and confirmatory assay were considered positive in the determination of infection prevalence.

### *Culicoides* community composition

The community composition was compared for each site by reporting the species richness [30]. Rarefaction curves were created in R studio version 1.0.143 using the iNEXT package version 2.0.15 [31] to understand the weight of sampling size in the apparent species richness at each site.

## Results

### *Culicoides* species identification

**Fig. 2:**
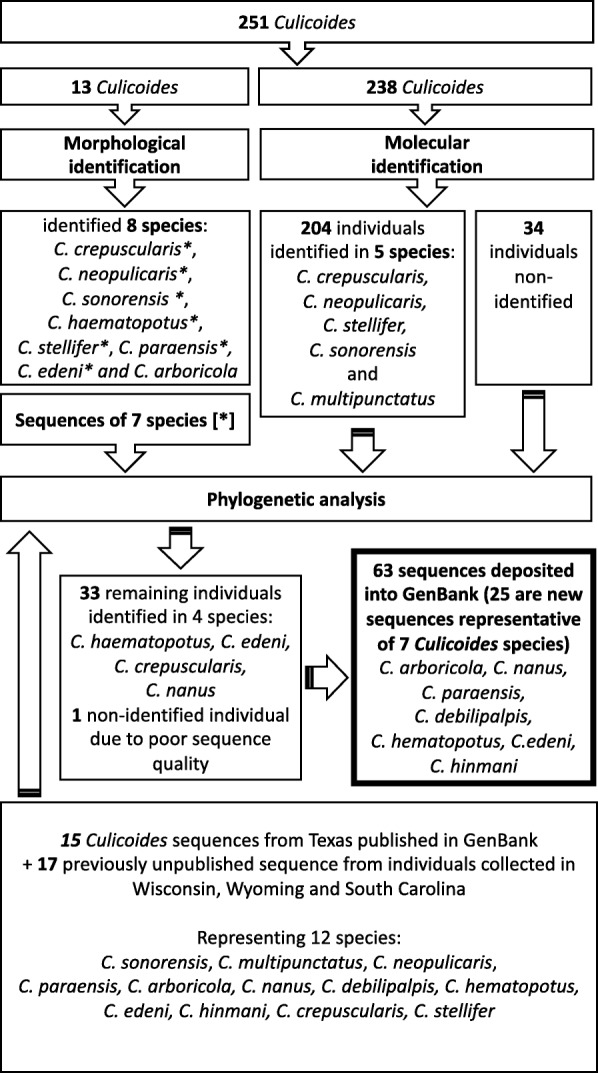
Flowchart of *Culicoides* morphological and molecular identification process

A total of 251 *Culicoides* were collected among the four sites. *Culicoides* communities were characterized using three different approaches: integration of morphological and molecular characteristics, molecular characteristics only, and phylogenetic analysis (Fig. 2). Thirteen individuals were set aside for morphological identification coupled with molecular analysis. Using morphological identification, eight species were identified from the 13 samples: *C. crepuscularis* Malloch, 1915; *C. neopulicaris* Wirth, 1955; *C. stellifer* (Coquillett, 1901); *C. haematopotus* Malloch, 1915; *C. sonorensis* Wirth & Jones, 1957; *C. paraensis* (Goeldi, 1905); *C edeni* Wirth & Blanton, 1974; and *C. arboricola* Root & Hoffman, 1937. Characteristics wing markings can be seen in Additional file 2: Figure S1. Because we use a non-destructive dissection and extraction technique, we were able to recover DNA from these individuals that were then subject to our PCR assay targeting the *cox*1 gene. DNA sequences from specimens representative of these *Culicoides* species (Additional file 3: Figure S2) were used later in the phylogenetic analysis with the exception of *C. arboricola* from which no sequences were recovered.

The 238 remaining samples were molecularly assessed, and we identified five species based on the *cox*1 gene. A BLAST search of NCBI GenBank database inferred the identity of the species as *C. crepuscularis* (*n* = 46, 99.2–100% identity), *C. neopulicaris* (*n* = 148, 99.2–100% identity), *C. stellifer* (*n* = 7, 95.6% identity), *C. sonorensis* (*n* = 1, 99.4% identity) and *C. multipunctatus* Malloch, 1915 (*n* = 1, 99.7% identity). Thirty-four sequences lacked sufficient sequence identity to infer species identification and one of the 34 sequences was removed from the analysis because of poor quality.

**Fig. 3:**
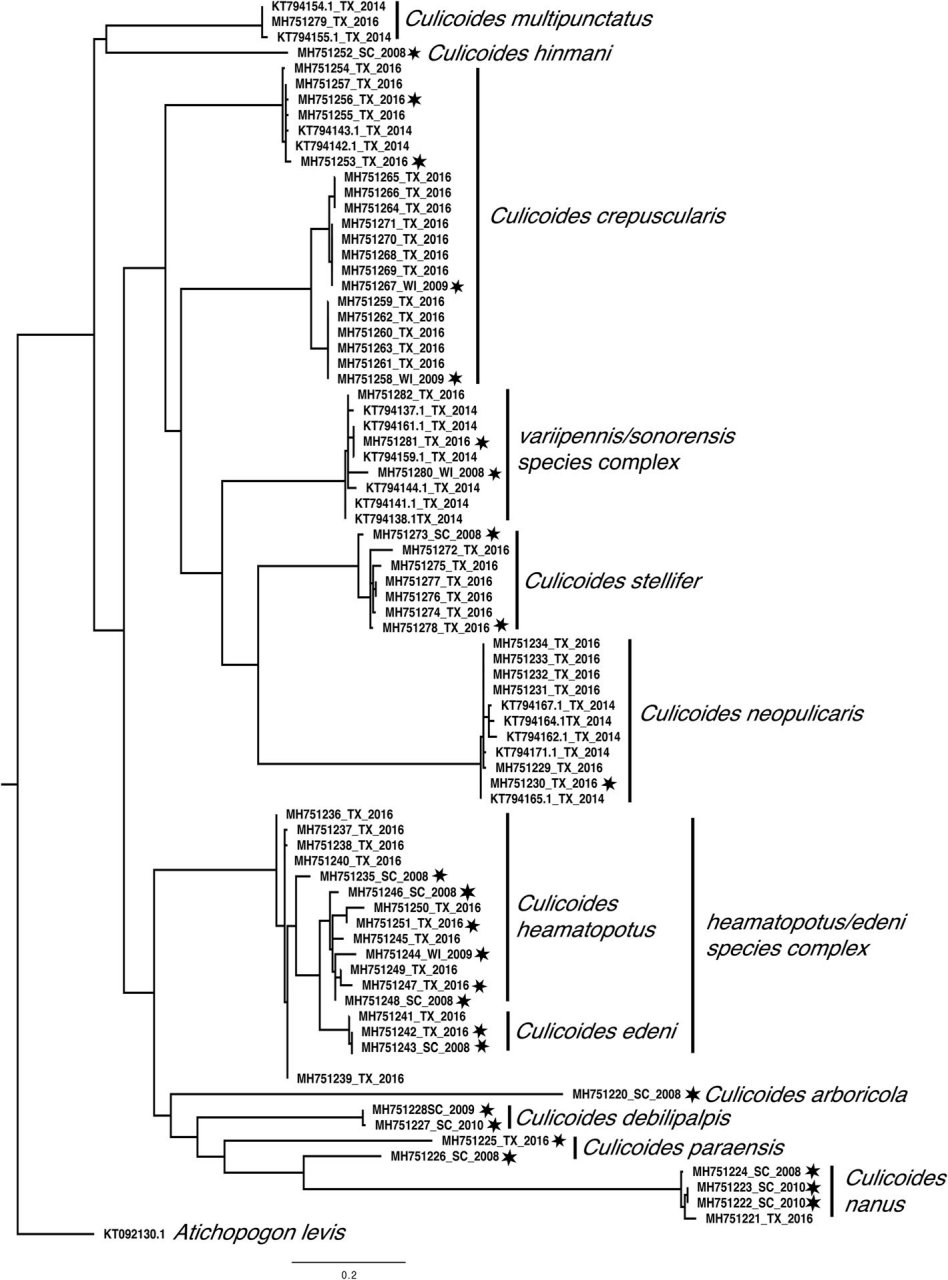
Phylogenetic relationship between *Culicoides* species based on a 405 bp region of the cytochrome *c* oxidase subunit 1 gene. *Culicoides* sequences from individuals captured in Texas (GenBank: MH751225, MH751229-34, MH751236-42, MH751245, MH751247, MH751249-51, MH751253-57, MH751259-66, MH751268-72, MH751274-79, MH751281-82), Winsconsin, Wyoming and South Carolina (GenBank: MH751220, MH751222-24, MH751226-28, MH751235, MH751243, MH751244, MH751246, MH751248, MH751252, MH751258, MH751267, MH751273, MH751280) are listed and individually marked with a star to indicate sample for which morphological characterization and molecular sequence were performed. The bootstrap support values above < 90% are shown at the corresponding nodes. Additionally, publically available *cox*1 sequences from Texas were added to the phylogenetic tree (GenBank: KT794137.1; KT794138.1; KT794141.1; KT794142.1; KT794143.1; KT794144.1, KT794154.1; KT794155.1; KT794159.1; KT794161.1; KT794162.1; KT794164.1; KT794165.1; KT794167.1; KT7941371.1). The tree was rooted to the *cox*1 sequence of *Atrichopogon levis* (GenBank: KT092130.1)

In order to identify the 33 remaining individuals, we performed a maximum likelihood analysis (Fig. 3) that included all the sequences obtained in this study 15 published sequences previously collected in Texas in 2015, and 17 otherwise unpublished North American *Culicoides* sequences from individuals collected between 2008 and 2010 in Wisconsin, Wyoming and South Carolina (Fig. 2) (GenBank: MH751220, MH751222-MH751224, MH751226-MH751228, MH751235, MH751243, MH751244, MH751246, MH751248, MH751252, MH751258, MH751267, MH751273, MH751280). All of our samples grouped in clades with known sequences and good bootstrap support, affording confidence of the species identity (Fig. 2). Two well-supported clades of *C. crepuscularis* (98% and 99% support) were observed, with one of them divided into two groups (82% and 95% support). Our sequences of *C. sonorensis* grouped together with *C. variipennis* and *C. sonorensis* with 99% support, but no further resolution between these species could be obtained using the *cox*1 gene. The sequences of *C. stellifer* grouped together in a well-supported clade (99% support). All *C. neopulicaris* grouped together with 100% support. *Culicoides haematopotus* and *C. edeni* were placed into a well-supported clade (96% support). Three groups were observed in this clade, with *C. edeni* (99% support), a group of eight *C. haematopotus* (90% support), and an unresolved second group of *C. haematopotus* (76% support). All *C. nanus* Root & Hoffman, 1937 grouped together in a single clade with 100% support, as did all *C. multipunctatus* (100% support). The *C. paraensis* collected from South Carolina did not group with the sequence of *C. paraensis* from this study. The 33 unknown sequences grouped within the well supported groups described above with one sequence attributed to *C. edeni*, 19 sequences to *C. hematopotus* and 13 sequences to *C. crepuscularis*. To simplify the phylogenetic tree, only a few representative of each species was mapped on the tree (Fig. 3).

### *Culicoides* species community composition

**Fig. 4:**
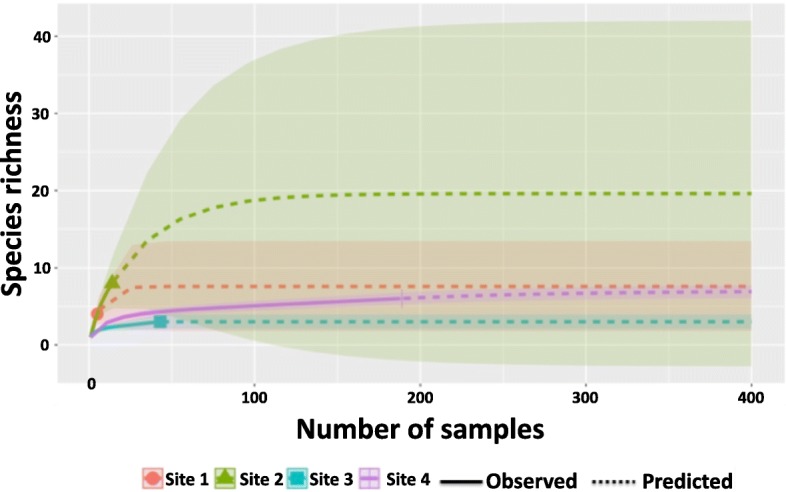
Rarefaction curves showing the influence of sample size on *Culicoides* species richness for each sampling site: Site 1 in red, Site 2 in green, Site 3 in blue and Site 4 in purple. Solid lines indicate the observed sample size and species richness whereas dashed lines represent the predicted sample size and species richness

Ten different species were present in the four trapping sites. The cumulative proportions of *Culicoides* collected from the four sites were principally *C. neopulicaris* (72.92%), *C. crepuscularis* (19.12%), *C. haematopotus* (3.98%) and *C. stellifer* (3.19%). On rare occasions (< 0.79%), *C. multipunctatus*, *C. sonorensis*, *C. arboricola*, *C. paraensis*, *C. nanus* and *C. edeni* were collected. Among individual sites (Table 1), species richness was the highest at site 2 with eight species. *Culicoides haematopotus* was the most abundant (28%), followed by *C. neopulicaris* (21%), *C. crepuscularis* (14%), *C. stellifer* (7%), *C. sonorensis* (7%), *C. paraensis* (7%), *C. edeni* (7%) and *C. nanus* (7%). Site 1 collections included *C. crepuscularis* (33.3%), *C. neopulicaris* (16.6%), *C. haematopotus* (16.6%), *C. arboricola* (16.6%) and *C. sonorensis* (16.6%). At site 4, the highest number of specimens were collected (*n* = 189) and represented five species (S = 5). *Culicoides neopulicaris* was the dominant species (73%) followed by *C. crepuscularis* (14%), *C. haematopotus* (8%), *C. stellifer* (4%), *C. multipunctatus* (1%) and *C. edeni* (1%). Site 3 had the lowest species richness (S = 3). The rarefaction analysis showed that the sampling effort in sites 3 and 4 was sufficient in explaining the richness of *Culicoides* present in these sites, whereas the sampling effort in sites 1 and 2 most likely resulted in an underestimation of the species richness (Fig. 4).

**Table 1:** *Culicoides* species composition and richness in four sites in College Station, Texas, 2016

Species	Site 1 *n* (%)	Site 2 *n* (%)	Site 3 *n* (%)	Site 4 *n* (%)
*C. paraensis* ^b^	0 (0)	1 (7)	0 (0)	0 (0)
*C. edeni* ^b,c^	0 (0)	1 (7)	0 (0)	1 (1)
*C. crepuscularis* ^b,c^	2 (33)	2 (14)	33 (77)	26 (14)
*C. multipunctatus* ^a^	0 (0)	0 (0)	0 (0)	1 (1)
*C. nanus* ^b,c^	0 (0)	1 (7)	0 (0)	0 (0)
*C. neopulicaris* ^b,c^	1 (16.6)	3 (21)	9 (21)	138 (73)
*C. sonorensis* ^b^	1 (16.6)	1 (7)	0 (0)	0 (0)
*C. stellifer* ^b^	0 (0)	1 (7)	0 (0)	7 (4)
*C. arboricola* ^d^	1 (16.6)	0 (0)	0 (0)	0 (0)
*C. haematopotus* ^b,c^	1 (16.6)	4 (28)	1 (2)	16 (8)
No. of specimens trapped (*N*)	6	14	43	189
Species richness (S)	5	8	3	5

^a^Molecular only

^b^Molecular and morphological

^c^Using phylogenetic analysis

^d^Morphological only

### Haemoparasite testing

One *C. crepuscularis* individual from site 3 tested positive for filarial nematodes with a sequence 100% similar to Onchocercidae gen. sp. isolated from common grackles (*Q. quiscala*) in Chicago (GenBank: JQ867040) [32]. The prevalence of this parasite in *C. crepuscularis* was 2.1%. One *C. crepuscularis* individual from site 3 tested positive for Haemosporida with a sequence 100% similar to *Haemoproteus sacharovi* isolated from a mourning dove in Arizona, USA (GenBank: KY653811, lineage hMODO1). Similarly, its prevalence in *C. crepuscularis* was 2.1%.

## Discussion

Among the 151 *Culicoides* spp. described from the Nearctic region, north of Mexico, 40 are reported in Texas, USA [33–35]. This study documents the presence of ten *Culicoides* spp. in an urban region of east central Texas during summer 2016. The dominant species in our urban study sites were *C. neopulicari*s, *C. crepuscularis*, *C. stellifer* and *C. haematopotus.* This study illustrates the benefit of integrated morphological and molecular systematics for accurate characterization of *Culicoides* communities [36]. All *Culicoides* spp. reported in this study, other than *C. edeni*, are known to occur in Texas (Shuts PT, unpublished data; [17]) and many species were likely undetected due to different factors including the short sampling period, small sample area, limited habitat coverage and the collection method.

*Culicoides neopulicari*s has been recorded previously in Texas and Louisiana [34], but very little is known about the larval habitats, feeding behavior and vector status of this species. *Culicoides crepuscularis*, the second most abundant species in this study, is one of the most abundant species in North America [34, 37]. Here we document *C. crepuscularis* infected with *Haemoproteus* DNA and filarial nematode DNA. *Culicoides crepuscularis* is a known vector of *H. danilewski* and *H. fringillae* found in Passeriformes [38, 39]. The cycle of transmission of *H. sacharovi* involves the hippoboscid fly *Pseudolynchia canariensis* as a vector [40]. However, *Culicoides* have also been suggested as potential vectors [41], and our positive test of a single individual shows that there is vector-parasite contact. We also found evidence of infection with a filarial nematode from the family Onchocercidae in *C. crepuscularis.* Our sequence shares 100% homology with a sequence of *Chandlerella quiscali* previously isolated from a common grackle (*Q. quiscala*) in Chicago. The common grackle was reported infected by *Ch. quiscali* with members of the family Ceratopogonidae suggested as vectors [14]. Additionally, *C. crepuscularis* is a known vector of filarial nematodes including *Ch. quiscali* [14], *Eufilaria* and *Splendidofilaria picacardina longicaudata* (Hibler C, unpublished data). Based on abundance and a clear capacity to transmit a number of parasites, future studies should focus on the role of *C. crepuscularis* in disease transmission in urban systems. Our results also report several *C. stellifer*, a widespread species throughout most of the USA, previously recorded infected with BTV [42], vesicular stomatitis virus [43] and West Nile virus [44]. Additionally, *C. haematopotus* was present at certain sites in College Station, Texas. This species is primarily ornithophilic but has been reported to also feed on some mammals [17, 37, 44–46] and can transmit multiple parasites including *Ch. quiscala* [14] and *Ch. striatospicula* as well as *E. longicaudata* (Hibler C, unpublished data) and *H. meleagridis* [47].

Our study contributes 63 sequences for both under-represented species in GenBank including *C. neopulicaris*, *C. crepuscula*ris, *C. stellifer*, *C. multipunctatus*, *C. sonorensis* and seven species unrepresented until our study in GenBank: *C. arboricola*, *C. nanus*, *C. debilipalpis* Lutz, 1913, *C. paraensis*, *C. haematopotus*, *C.edeni* and *C. hinmani* Khalaf, 1952. Our phylogenetic analysis based on the *cox*1 gene revealed well-supported clusters which were in agreement with the morphological determination with the exception of *C. multipunctatus* for which no morphological identification was performed. While most of the species characterized fall into distinct clades (e.g*. C. stelliffer*, *C. neopulicaris*, *C. nanus*, *C. multipunctatus* and *C. paraensis*), *C. crepuscularis* fell into two distinct clades, one of which was divided into two distinct groups. This observation suggests the presence of cryptic species within *C. crepuscularis*. Like many studies, our molecular analysis grouped *C. variipennis* and *C. sonorensis* into a single clade. Morphologically these two species are distinguishable [48], but the molecular analysis using the *cox*1 marker groups them in a single clade (Shults PT, unpublished data; [49]). *Culicoides sonorensis* is a known vector of BTV, EHDV and Main Drain virus [50–54]. Using molecular and morphological characteristics, we were able to separate *C. edeni* (*n* = 2) from *C. haematopotus* (*n* = 14) [37] (Fig. 3). *Culicoides edeni* is a species of interest due to its involvement in the transmission of *H. danilewskyi* in blue jays [55]. To our knowledge, this study represents the first record of *C. edeni* in Texas. Prior to this study, *C. edeni* was known from The Bahamas, Florida, and South Carolina (Swanson DA, unpublished data; [37]). Due to the morphological similarities, multiple genetic clades, and disjointed distribution, there is potential for undescribed cryptic species to exist within a *C. edeni*-*C. haematopotus* complex; species for which we know little from an epidemiological standpoint.

Among the other species found in our study, two are known vectors of pathogens. *Culicoides paraensis* is a species that preferentially breeds in tree holes [56] and feeds on a variety of birds and mammals, including humans [45, 56–58], and are known vector for *Mansonella ozzardi* [59] and *Oropouche* orthobunyavirus [60] in South America and the Caribbean. This is only the second record of *C. paraensis* in Texas. Our sequence of *C. paraensis* did not group with *C. paraensis* from South Carolina (82.28% nucleotide identity) highlighting possible cryptic species diversity, variation in *cox*1 sequences due to geographical location or a potential misidentification that will require further investigation. However, this situation exemplifies the need for integrated taxonomic approaches to species identification. The sequences of both of these specimens were obtained with non-destructive methods, and therefore reexamination of these two individuals is possible.

*Culicoides multipunctatus* is common and abundant throughout central and south Texas (Shults PT, unpublished data; Schoenthal C, unpublished data; [17]). Its rarity in this study could offer insight into the seasonal distribution of this species as well as the unsuitability of the habitat. Finally, we detected *C. nanus*, a species that was previously recorded in Texas [17, 61] but nothing is known of the biting records or vector capacity of this species [37].

## Conclusions

We document the abundance of ten *Culicoides* spp. in an urban environment in Texas, USA. The identification of these species was enhanced by an integrative taxonomic approach, without which some species could have been misidentified when using morphological or molecular techniques only. Molecular barcoding sequences were submitted to GenBank for all species, including seven of which did not have previous sequence data in the GenBank database. One species, *C. crepuscularis*, was found to be positive for Onchocercidae gen. sp. and *Haemoproteus sacharovi*, supporting that they may be involved in the transmission of these avian parasites. Further investigations on vector competence of *C. crepuscularis* with *H. sacharovi* and filarial nematodes, is needed to support for this hypothesis.

## Additional files

Additional file 1: **Table S1.** Primer sequences for PCR. (DOCX 29 kb)

Additional file 2: **Figure S1.** Wing markings of the eight morphologically identified *Culicoides* species collected in College Station. A, *C. arboricola*; B, *C. crepuscularis*; C, *C. edeni*; D, *C. haematopotus*; E, *C. neopulicaris*; F, *C. paraensis*; G, *C. sonorensis*; H, *C. stellifer*. (TIF 15482 kb)

Additional file 3: **Figure S2.** Chromatogram presenting the sequence of *cox*1 gene for each of the seven *Culicoides* species collected in College Station and morphologically identified: A, *C. crepuscularis*; B, *C. edeni*; C, *C. haematopotus*; D, *C. neopulicaris*; E, *C. paraensis*; F, *C. sonorensis*; G, *C. stellifer*. On the left panel are the chromatographs representing sequence generated with the forward primer and on the right panel the chromatographs associated with the sequence generated with the reverse sequence. (TIFF 36312 kb)

## Abbreviations

BTV: Bluetongue virus
*cox*1: cytochrome *c* oxidase subunit 1
EHD: Epizootic hemorrhagic disease
