# Supplementary material for: Culicoides species community composition and infection status with parasites in an urban environment of east central Texas, USA
Source: Parasit Vectors. 2019 Jan 16;12:39. doi: 10.1186/s13071-018-3283-9 (PMC6335769; doi:10.1186/s13071-018-3283-9)
Supplement: Supplementary file 1 — Table S1. Primer sequences for PCR. (DOCX 29 kb) [file 13071_2018_3283_MOESM1_ESM.docx]

**Additional file 1: Table S1.** Primer sequence for PCR

| **Organism** | **Gene** | **Forward primer sequence (5’-3’)** | **Reverse Primer sequence (5’-3’)** | **Amplicon Size (bp)** | **Reference** |
| --- | --- | --- | --- | --- | --- |
| Metazoan invertebrates | CO1 | LCO1490  GGTCAACAAATCATAAAGATATTGG | HC02198  TAAACTTCAGGGTGACCAAAAAATCA | 658 | [1] |
| Filarial nematodes | 18S | ChandFO  GAGACCGTTCTCTTTGAGGCC | ChandRO GTCAAGGCGTANNTTTACCGCCGA | 580 | [2] |
|  | CO1 | COIintF  TGATTGGT GGTTTTGGTAA  COIintF  TGATTGGT GGTTTTGGTAA | COIintR  ATAAGTACGAGTATCAATATC  COIintRn CATAAAAAGAAGTATTAAAATTACG | 688  340 | [3, 4] |
| Haemosporida | 16S | 343F  GCTCACGCATCGCTTCT | 496R  GACCGGTCATTTTCTTTG | 154 | [5, 6] |
|  | Cyt b | 3932F  GGGTTATGTATTACCTTGGGGTC  413F  GTGCAACYGTTATTACTAA | DW4  TGTTTGCTTGGGAGCTGTAATCATAATGTG  926R  CATCCAATCCATAATAAAGCAT | 702  513 | [5, 7, 8] |

**References**

1. Folmer O, Black M, Hoeh W, Lutz R, Vrijenhoek R: **DNA primers for amplification of mitochondrial cytochrome c oxidase subunit I from diverse metazoan invertebrates**. *Mol Mar Biol Biotechnol* 1994, **3**(5):294-299.

2. Hamer GL, Anderson TK, Berry GE, Makohon-Moore AP, Crafton JC, Brawn JD, Dolinski AC, Krebs BL, Ruiz MO, Muzzall PM *et al*: **Prevalence of filarioid nematodes and trypanosomes in American robins and house sparrows, Chicago USA**. *International journal for parasitology Parasites and wildlife* 2013, **2**:42-49.

3. Bataille A, Fournie G, Cruz M, Cedeno V, Parker PG, Cunningham AA, Goodman SJ: **Host selection and parasite infection in *Aedes taeniorhynchus*, endemic disease vector in the Galapagos Islands**. *Infect Genet Evol* 2012, **12**(8):1831-1841.

4. Casiraghi M, Anderson TJ, Bandi C, Bazzocchi C, Genchi C: **A phylogenetic analysis of filarial nematodes: comparison with the phylogeny of *Wolbachia* endosymbionts**. *Parasitol* 2001, **122** (1):93-103.

5. Fecchio A, Lima MR, Svensson-Coelho M, Marini MA, Ricklefs RE: **Structure and organization of an avian haemosporidian assemblage in a Neotropical savanna in Brazil**. *Parasitol* 2013, **140**(2):181-192.

6. Fallon SM, Ricklefs RE, Swanson BL, Bermingham E: **Detecting avian malaria: an improved polymerase chain reaction diagnostic**. *J Parasitol* 2003, **89**(5):1044-1047.

7. Perkins SL, Schall JJ: **A molecular phylogeny of malarial parasites recovered from cytochrome b gene sequences**. *J Parasitol* 2002, **88**(5):972-978.

8. Ricklefs RE, Swanson BL, Fallon SM, MartÍnez-AbraÍn A, Scheuerlein A, Gray J, Latta SC: **Community relationships of avian malaria parasites in southern Missouri**. 2005, **75**(4):543-559.
