# Supplementary figures and images for: Culicoides species community composition and infection status with parasites in an urban environment of east central Texas, USA
Source: Parasit Vectors. 2019 Jan 16;12:39. doi: 10.1186/s13071-018-3283-9 (PMC6335769; doi:10.1186/s13071-018-3283-9)

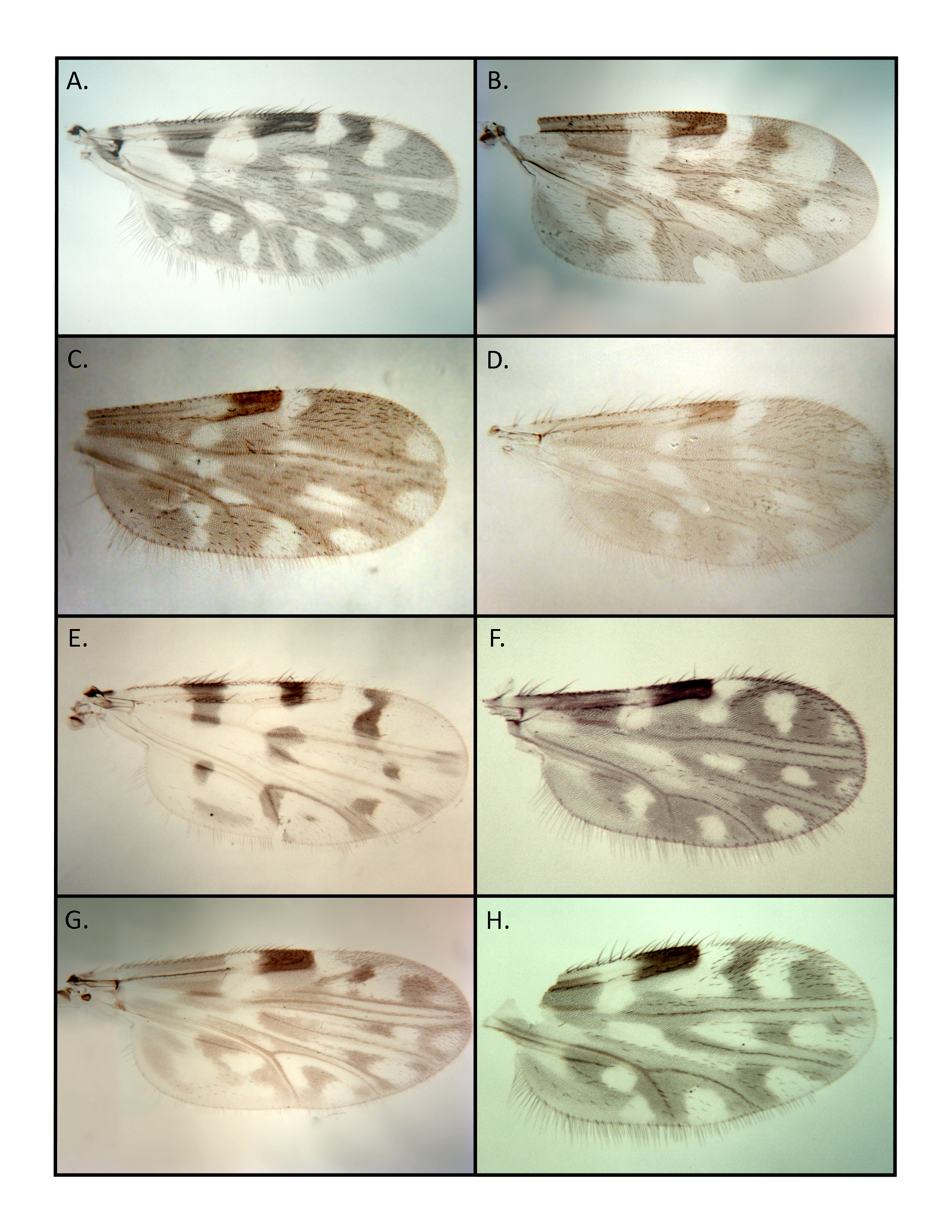

Supplement: Supplementary file 2 — Figure S1. Wing markings of the eight morphologically identified Culicoides species collected in College Station. A, C. arboricola; B, C. crepuscularis; C, C. edeni; D, C. haematopotus; E, C. neopulicaris; F, C. paraensis; G, C. sonorensis; H, C. stellifer. (TIF 15482 kb) [file 13071_2018_3283_MOESM2_ESM.tif]

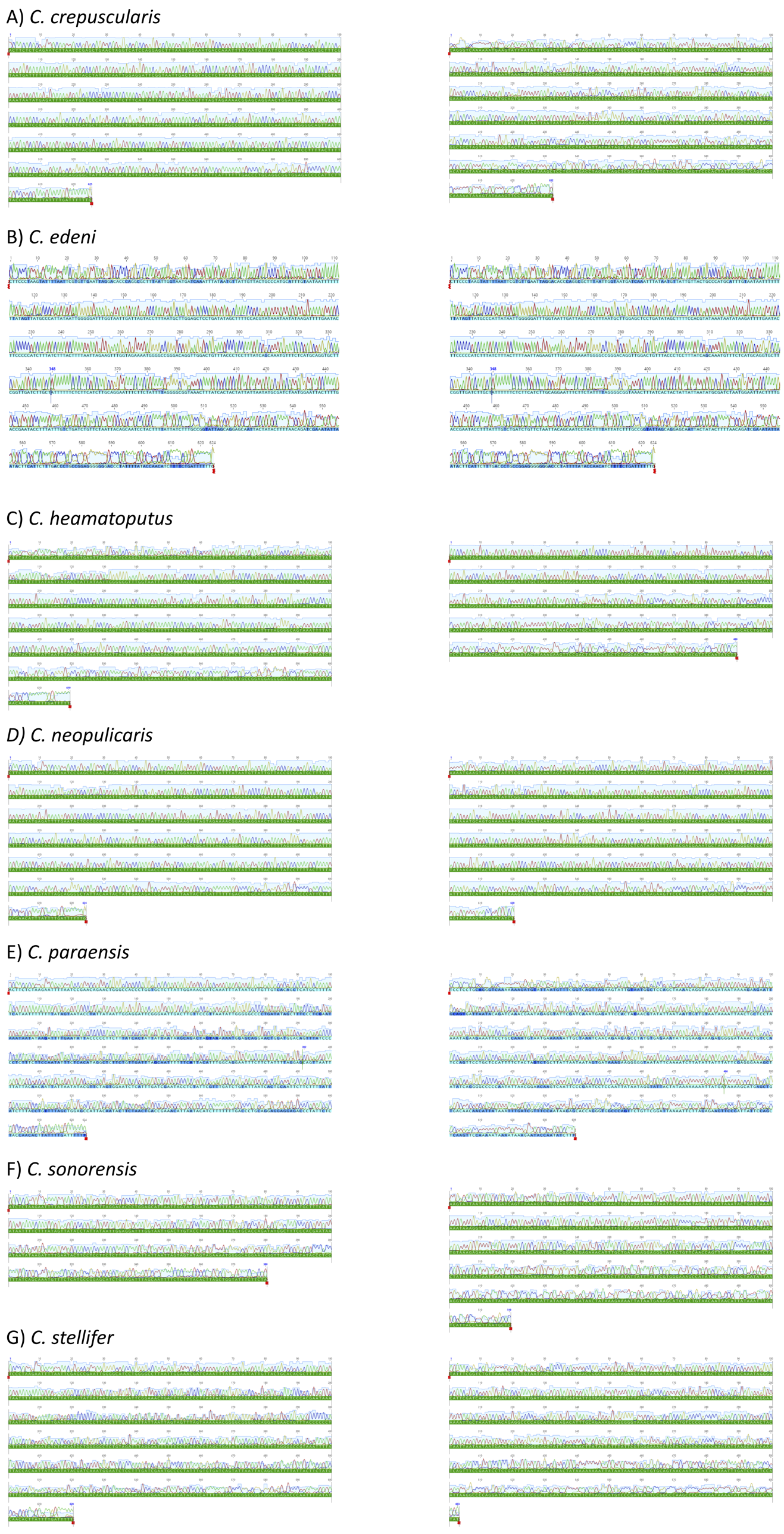

Supplement: Supplementary file 3 — Figure S2. Chromatogram presenting the sequence of cox1 gene for each of the seven Culicoides species collected in College Station and morphologically identified: A, C. crepuscularis; B, C. edeni; C, C. haematopotus; D, C. neopulicaris; E, C. paraensis; F, C. sonorensis; G, C. stellifer. On the left panel are the chromatographs representing sequence generated with the forward primer and on the right panel the chromatographs associated with the sequence generated with the reverse sequence. (TIFF 36312 kb) [file 13071_2018_3283_MOESM3_ESM.tiff]
